# Antimicrobial de-escalation in patients with high-risk febrile neutropenia: Attitudes and practices of adult hospital care providers

**DOI:** 10.1017/ash.2021.185

**Published:** 2021-08-19

**Authors:** Chelsea A. Gorsline, Milner B. Staub, George E. Nelson, Whitney J. Nesbitt, Bhagirathbhai R. Dholaria, Gowri Satyanarayana

**Affiliations:** 1 Division of Infectious Diseases, Department of Medicine, Vanderbilt University Medical Center, Nashville, Tennessee; 2 Department of Pharmaceutical Services, Vanderbilt University Medical Center, Nashville, Tennessee; 3 Division of Hematology and Oncology, Department of Medicine, Vanderbilt University Medical Center, Nashville, Tennessee

**Keywords:** Antimicrobial stewardship, febrile neutropenia, malignant hematology, stem cell transplant, early de-escalation of antimicrobials

## Abstract

In a survey of adult hospital providers regarding antibiotic use in the treatment of febrile neutropenia, clinical fellows, and pharmacists showed higher comfort levels with early antimicrobial de-escalation compared to hematology-oncology and transplant infectious diseases physicians. These frontline team members are ideal partners to champion antimicrobial stewardship interventions in febrile neutropenia.

Although antimicrobial stewardship programs (ASPs) are widespread, implementation of interventions for patients with hematologic malignancies and hematopoietic stem-cell transplantation (HCT) are lacking. These patients are prone to high-risk febrile neutropenia requiring treatment with broad-spectrum intravenous antibiotics. Current Infectious Diseases Society of America (IDSA) guidelines published in 2010 recommend the continuation of broad-spectrum antibiotics until absolute neutrophil count (ANC) recovery (>500 cells/mm^3^).^1^ However, the European Conference on Infections in Leukemia (ECIL) guidelines published in 2013 recommend early antimicrobial de-escalation in stable patients.^2^ Subsequent studies showed the safety and efficacy of this approach,^3–5^ but early de-escalation has been inconsistently adopted by US institutions,^6,7^ and it is not currently considered the standard of care.

At our institution, the hematology-oncology (HO) unit administers the most inpatient antimicrobials; however, an early de-escalation protocol for febrile neutropenia, recently developed by our ASP to reduce antibiotic use, had limited adoption among providers. Therefore, we developed and distributed a knowledge, attitudes and practices (KAP) survey to key staff to understand more fully barriers to adoption of the protocol at our institution. Here, we describe the survey results and steps to improve protocol adherence.

## Methods

This single-center study was approved by the Vanderbilt University Medical Center (VUMC) Institutional Review Board. We developed a 15-question, peer-reviewed, electronic survey that was e-mailed to HO attending physicians, HO clinical fellows, HO midlevel providers, transplant infectious diseases (TID) attending physicians, and HO and ID pharmacists (PharmD) at VUMC in September 2020. All responses were anonymous.

The surveys consisted of multiple choice and Likert-scale questions assessing respondents’ comfort level with antimicrobial de-escalation and preferred methods of antibiotic use guidance (Supplement 1 online). Two clinical scenarios describing an adult patient with hematologic malignancy and high-risk febrile neutropenia with either a known source of infection or no known infectious source were presented to gauge provider comfort with antimicrobial de-escalation and to identify the most important factor when considering de-escalation. Comfort level was assessed using a 4-point Likert scale (not comfortable, somewhat comfortably, comfortable, very comfortable). Respondents chose the most important factor affecting antimicrobial de-escalation decisions from the following items: adverse event from antimicrobial, multidrug-resistant organism (MDRO) emergence, morbidity/mortality, following guidelines, and severity of illness. Respondents selected the ASP resource(s) that would best aid with de-escalation of antimicrobials from the following list: electronic resource, decision support in the medical record, automated antibiotic use report, real-time antibiotic use dashboard or in-person handshake rounds with a member of ASP.

Surveys with incomplete responses were excluded. Responses were analyzed by demographic group. Descriptive analyses were performed, and absolute and percent frequencies were tabulated for each variable. For questions utilizing a Likert scale, responses were analyzed using a proportional odds model. Analyses were performed using Excel version 16.48 software (Microsoft, Redmond, WA) and STATA/MP version 16.1 software (StataCorp, College Station, TX).

## Results

### Respondents

The survey was e-mailed to 112 participants, of whom 47 (42%) responded. Response rates within each specialty were as follows: HO (n = 35 of 99, 36%), TID (n = 4 of 5, 80%), HO PharmD (n = 4 of 5, 80%), and ID PharmD (n = 2 of 3, 67%). Two surveys were omitted due to incomplete responses; thus, 45 surveys were included for analysis (40%). Of the 45 respondents in the final cohort, 35 (78%) were HO; 4 (9%) were TID, 4 (9%) were HO PharmD, and 2 (4%) were ID PharmD (n = 2 of 45, 4%). Also, 29 respondents (64%) reported 0–5 years of experience, 8 (18%) had 6–10 years of experience, and 8 (18%) had >10 years of experience.

### Clinical scenarios

For each scenario, proportional odds ratios for comfort level with antimicrobial de-escalation were calculated (Table 1). In the clinical scenario in which an infection was identified, TID were nearly 5 times more likely to feel comfortable de-escalating antimicrobials than HO attending physicians, clinical fellows, and midlevel providers (OR, 4.99; 95% CI, 0.78–31.97). Compared to providers with 0–5 years of experience, those with 6–10 years of experience (OR, 1.44; 95% CI, 0.26–7.87) and >10 years of experience (OR, 1.90; 95% CI, 0.48–7.43) had higher odds of feeling comfortable de-escalating antimicrobials. HO clinical fellows were more likely to feel comfortable de-escalating antimicrobials than attending physicians (OR, 1.11; 95% CI, 0.23–5.30). In the clinical scenario in which no infection was identified, HO PharmD were more likely to feel comfortable de-escalating antimicrobials than HO attending physicians, clinical fellows and midlevel providers (OR, 6.91; 95% CI, 0.87–53.33). Pharmacists had the highest odds of feeling comfortable de-escalating antimicrobials compared to attending physicians (OR, 3.46; 95% CI, 0.51–23.33). No comparison in any of these scenarios reached statistical significance.

**Table 1.** Proportional Odds Ratios for Variables in Each Clinical Scenario

Question Domain	Odds Ratio (95% Confidence Interval)	
	Clinical Scenario Known Infection	Clinical Scenario No Known Infection
**Specialty**		
Hematology/Oncology	Reference	Reference
Transplant infectious diseases	4.99 (0.78–31.97)	2.78 (0.49–15.76)
Hematology/Oncology pharmacist	0.62 (0.09–4.27)	6.91 (0.89–53.33)
Infectious diseases pharmacist	0.46 (0.01–28.61)	1.06 (0.02–56.05)
**Years of experience**		
0–5 y of experience	Reference	Reference
6–10 y of experience	1.44 (0.26–7.87)	3.45 (0.63–18.83)
>10 y of experience	1.90 (0.48–7.43)	1.54 (0.33–7.04)
**Service level**		
Attending	Reference	Reference
Fellow	1.11 (0.23–5.30)	0.46 (0.09–2.21)
Nurse practitioner	0.74 (0.18–3.04)	0.88 (0.19–3.96)
Physician assistant	0.29 (0.02–3.52)	1.20 (0.09–15.33)
Pharmacist	0.43 (0.06–2.79)	3.46 (0.51–23.33)

### Medical decision making

**Fig. 1.**
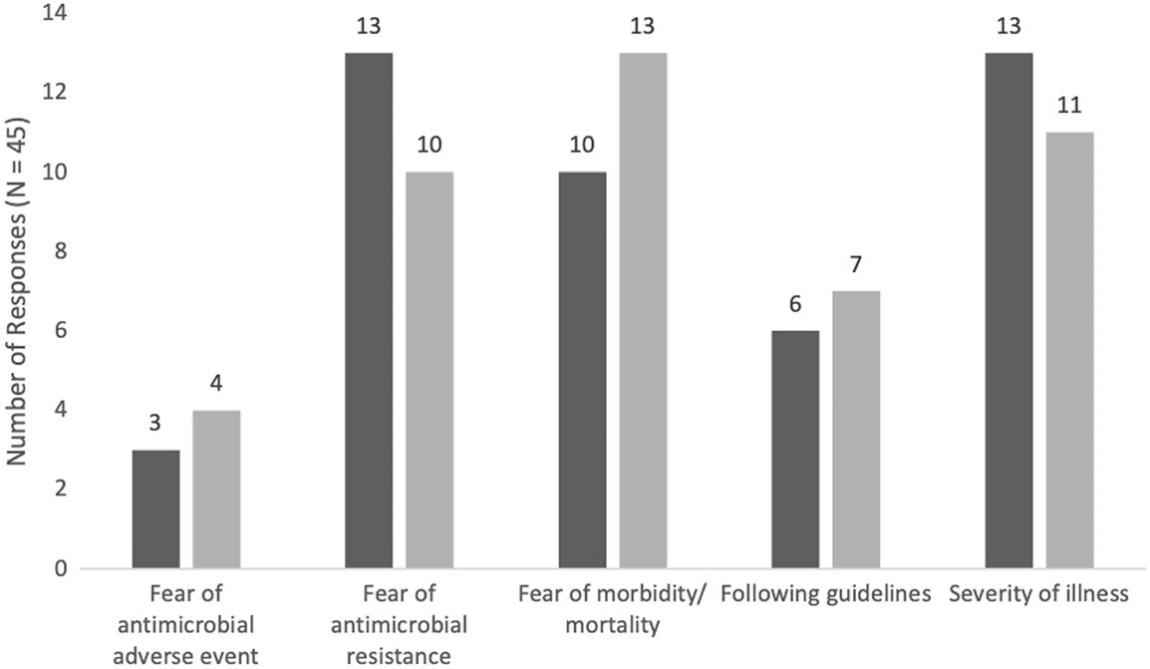
Aggregate response rates of the most important factor when considering antimicrobial de-escalation for each clinical scenario.

In the clinical scenario in which an infection was identified, the most important factors influencing antimicrobial de-escalation were fear of developing an MDRO infection (n = 13 of 45, 29%), severity of illness (n = 13 of 45, 29%), and fear of morbidity and mortality (n = 10 of 45, 22%). In the clinical scenario in which no source of infection was identified, the same 3 responses were chosen: fear of morbidity and mortality (n = 13 of 45, 29%), severity of illness (n = 11 of 45, 24%), and fear of developing MDRO infection (n = 10 of 45, 22%) (Fig. 1).

### Preferred antibiotic stewardship program resource

Among the 45 respondents, 82 ASP resources were identified as most helpful to guide antimicrobial de-escalation in high-risk febrile neutropenia. The top 3 choices were in-person handshake rounds (n = 32 of 82, 39%), locally developed electronic resources (n = 28 of 82, 34%), and decision support built into the medical record (n = 13 of 82, 16%).

## Discussion

To our knowledge, this is the first survey evaluating antimicrobial de-escalation in high-risk febrile neutropenia that included HO, TID, ID PharmD, and HO PharmD providers. Although none of the proportional odds models achieved statistical significance, 2 interesting trends emerged: (1) HO clinical fellows were the most comfortable de-escalating antimicrobials when an infection was identified as the cause of fever and (2) pharmacists were the most comfortable when there was no infection identified. The finding of house staff having more comfort with de-escalation was surprising because in the same scenario, providers with more years of experience showed higher levels of comfort with de-escalation. However, prior studies have shown house staff, as well as nonstewardship pharmacists, to be advantageous targets for ASP interventions.^8–10^ The higher comfort levels among these groups at our institution may reflect more willingness to adopt our locally developed febrile neutropenia antimicrobial de-escalation protocol.

Many respondents cited MDROs as influencing antimicrobial decisions but few cited antimicrobial adverse events or guideline adherence. Rather, respondents were driven by fear of poor patient outcomes, highlighting provider anxiety as a major barrier to de-escalation. Many respondents desired ASP guidance in a handshake rounds format, which suggests that antimicrobial de-escalation may be achieved through personalized antibiotic use feedback and coaching. The limitations of this study include its small sample size and single-center design.

The results of this study will be used to create a multidisciplinary de-escalation team that includes non-ASP pharmacists, house staff, and midlevel providers in addition to TID and ASP at our institution. This partnership will train new ASP champions who can then encourage use of the de-escalation protocol among other providers who are less comfortable with this practice. The de-escalation team will also provide tailored antibiotic use feedback, counter fears about patient complexity, and educate about the risks of antimicrobial adverse events and resistance. Future studies will assess outcomes of implementing a de-escalation team.

## Supplementary material

For supplementary material accompanying this paper visit https://doi.org/10.1017/ash.2021.185. click here to view supplementary material
