# Supplementary material for: Antimicrobial de-escalation in patients with high-risk febrile neutropenia: Attitudes and practices of adult hospital care providers
Source: Antimicrob Steward Healthc Epidemiol. 2021 Aug 19;1(1):e22. doi: 10.1017/ash.2021.185 (PMC9495534; doi:10.1017/ash.2021.185)
Supplement: Supplementary file 1 [file S2732494X21001856sup001.docx]

**Febrile Neutropenia Survey**

Current guidelines demonstrate variable recommendations for antibiotic use in the management of febrile

neutropenia. This survey explores your knowledge, attitudes and practices in the use of antibiotics for high-risk

malignant hematology patients admitted to the hospital with febrile neutropenia. For the purpose of this survey,

febrile neutropenia is defined as an absolute neutrophil count (ANC) less than 500 and temperature greater than or equal to 100.4F.

**Demographics**

What is your medical subspecialty?

- Hematology/Oncology
- Transplant Infectious Diseases
- Hematology/Oncology Clinical Pharmacist
- Infectious Diseases Clinical Pharmacist

What is your service level?

- Attending Physician
- Senior Fellow (PGY-5 and above)
- Nurse Practitioner
- Physician Assistant
- Pharmacist

How many years have you been in practice (after completion of your education)?

- 0 - 5 years
- 6 - 10 years
- Greater than 10 years

**Consider the possible IATROGENIC complications that may occur in a patient with a**

**hematologic malignancy and febrile neutropenia who is admitted to the hospital.**

**Please rank each option in order from most important (1) to least important (7) in determining**

**overall patient outcome.**

- Rank: ­­­­­­­­­­­­­­­____________ C diff associated diarrhea
- Rank: ­­­­­­­­­­­­­­­____________ Catheter associated thrombosis
- Rank: ­­­­­­­­­­­­­­­____________ Catheter associated urinary tract infection (CAUTI)
- Rank: ­­­­­­­­­­­­­­­____________ Central line associated bloodstream infection (CLABSI)
- Rank: ­­­­­­­­­­­­­­­____________ Emergence of multi-drug resistant organism
- Rank: ­­­­­­­­­­­­­­­____________ Mild reaction to antimicrobial (drug fever, drug rash, etc)
- Rank: ­­­­­­­­­­­­­­­____________ Severe reaction to antimicrobial (anaphylaxis, DRESS, etc)

**Consider the possible INFECTIOUS complications that may occur in a patient with a**

**hematologic malignancy and febrile neutropenia who is admitted to the hospital.**

**Please rank each option in order from most important (1) to least important (7) in determining**

**overall patient outcome.**

- Rank: ­­­­­­­­­­­­­­­____________ Septic shock
- Rank: ­­­­­­­­­­­­­­­____________ Intensive care unit admission
- Rank: ­­­­­­­­­­­­­­­____________ Need for surgery or invasive procedure
- Rank: ­­­­­­­­­­­­­­­____________ Recurrent fever after antibiotics are discontinued
- Rank: ­­­­­­­­­­­­­­­____________ Recurrent fever while on broad spectrum antibiotics for treatment of febrile

neutropenia

- Rank: ­­­­­­­­­­­­­­­____________ Hospital days beyond ANC recovery
- Rank: ­­­­­­­­­­­­­­­____________ Re-hospitalization within 30 days

**Consider the possible outcomes that may occur in a patient with a hematologic malignancy**

**and febrile neutropenia who is admitted to the hospital.**

**Please rank each option in order from most important (1) to least important (7) in determining**

**overall patient outcome.**

- Rank: ­­­­­­­­­­­­­­­____________ Deconditioning with decline in performance status
- Rank: ­­­­­­­­­­­­­­­____________ DVT/PE
- Rank: ­­­­­­­­­­­­­­­____________ Malnutrition
- Rank: ­­­­­­­­­­­­­­­____________ Mucositis
- Rank: ­­­­­­­­­­­­­­­____________ Organ dysfunction (respiratory failure, MI, Afib, AKI, liver injury, etc)
- Rank: ­­­­­­­­­­­­­­­____________ Relapse of underlying disease
- Rank: ­­­­­­­­­­­­­­­____________ Typhilitis

**The following questions use the terms "ESCALATION" and "DE-ESCALATION" to describe**

**prescribing practices of antibiotics. For the purpose of this study, ESCALATION is defined as**

**the addition of antimicrobial agents or to the broadening of antimicrobial therapy.**

**DE-ESCALATION is defined as 1) narrowing of antimicrobial therapy, 2) step down of antibiotic**

**therapy to oral prophylaxis or 3) discontinuation of antibiotics altogether.**

**If you are a hematology/oncology provider, please answer these questions as if you are acting**

**independently of an Infectious Diseases consultant.**

Do you feel comfortable ESCALATING antimicrobial therapy in a patient whose ANC is less than 500 and is febrile

despite empiric antibiotics?

- Not comfortable
- Somewhat comfortable
- Comfortable
- Very comfortable

**Ms. B is a 53 year old woman with newly diagnosed acute lymphoblastic leukemia (ALL) who has undergone Cycle #1 of HyperCVAD. Five days after becoming neutropenic, she develops a fever to 102 F and is started on**

**Piperacillin-tazobactam. She continues to have fever for the next 24 hours, so Vancomycin is added to her regimen. Blood cultures drawn on the first day of fever and neutropenia grow viridans Streptococci. No other infectious source of fever is identified and her fevers resolve. Do you feel comfortable DE-ESCALATING antibiotics in this patient whose ANC is less than 500 when an infectious etiology of her fever has been identified?**

- Not comfortable
- Somewhat comfortable
- Comfortable
- Very comfortable

In the scenario above, a patient undergoes an extensive work-up for febrile neutropenia. An infectious cause of fever is identified and the patient's fever resolves. What factor is the most important when considering DE-ESCALATION of antibiotics in this patient who is still neutropenic?

- Fear of adverse event from use of antimicrobial(s)
- Fear of developing antimicrobial resistance
- Fear of increased morbidity or mortality
- Following guidelines
- Severity of illness

**Mr. J is a 58 year old man with newly diagnosed acute myeloid leukemia (AML) who has undergone 7+3 induction chemotherapy. Day 14 bone marrow biopsy shows residual disease. On hospital day 18, he remains neutropenic and has a fever to 101 F. An extensive work-up over a three-day period is negative, including: routine blood and urine cultures, Clostridium difficile toxin, computerized tomography (CT) scan of the sinuses, chest, abdomen, pelvis, and fungal disease evaluation. The patient's fevers resolve. Current antimicrobial therapy includes Cefepime, Vancomycin, and Micafungin. Do you feel comfortable DE-ESCALATING antibiotics in this patient whose ANC is less than 500 with NO known infectious cause of fever?**

- Not comfortable
- Somewhat comfortable
- Comfortable
- Very comfortable

In the scenario above, a patient undergoes an extensive work-up for febrile neutropenia. No infectious cause of fever is identified and the patient's fever resolves. What factor is the most important when considering DE-ESCALATION of antibiotics in this patient who is still neutropenic?

- Fear of adverse event from use of antimicrobial(s)
- Fear of developing antimicrobial resistance
- Fear of increased morbidity or mortality
- Following guidelines
- Severity of illness

**Does your institution have an antimicrobial de-escalation protocol for the management of febrile neutropenia?**

- Yes
- No
- Not sure

**I would feel the most comfortable de-escalating antibiotics if I had access to:**

**(You may select more than one option)**

- Electronic resource, such as a website with institutional guidance and an algorithm
- Decision support built into electronic medical record, such as an automated notification
- Automated report detailing my antimicrobial usage
- Real-time dashboard tracking my antimicrobial utilization
- Handshake rounds: in-person feedback with an Antimicrobial Stewardship pharmacist/physician team
